# Emerging Roles of Ceramides in Breast Cancer Biology and Therapy

**DOI:** 10.3390/ijms231911178

**Published:** 2022-09-23

**Authors:** Purab Pal, G. Ekin Atilla-Gokcumen, Jonna Frasor

**Affiliations:** 1 Department of Physiology and Biophysics, College of Medicine, University of Illinois at Chicago, Chicago, IL 60612, USA; 2 Department of Chemistry, University at Buffalo, The State University of New York (SUNY), Buffalo, NY 14260, USA

**Keywords:** ceramides, breast cancer, apoptosis, sphingolipids, drug resistance

## Abstract

One of the classic hallmarks of cancer is the imbalance between elevated cell proliferation and reduced cell death. Ceramide, a bioactive sphingolipid that can regulate this balance, has long been implicated in cancer. While the effects of ceramide on cell death and therapeutic efficacy are well established, emerging evidence indicates that ceramide turnover to downstream sphingolipids, such as sphingomyelin, hexosylceramides, sphingosine-1-phosphate, and ceramide-1-phosphate, is equally important in driving pro-tumorigenic phenotypes, such as proliferation, survival, migration, stemness, and therapy resistance. The complex and dynamic sphingolipid network has been extensively studied in several cancers, including breast cancer, to find key sphingolipidomic alterations that can be exploited to develop new therapeutic strategies to improve patient outcomes. Here, we review how the current literature shapes our understanding of how ceramide synthesis and turnover are altered in breast cancer and how these changes offer potential strategies to improve breast cancer therapy.

## 1. Introduction

With an estimated 287,850 new cases in 2022, breast cancer (BC) is the most common cancer in women (15% of all new cancer cases) [1]. The mortality in BC patients has decreased steadily over the last twenty years from 26.6% to 19.4% [1], which can be largely attributed to improved therapeutic strategies that have come from continuous progress in understanding breast tumor biology. Therefore, the identification and characterization of important molecular targets driving BC development and progression are essential to improve the therapeutic efficacies of existing treatments, as well as to develop new therapeutic strategies.

Over the past few decades, ceramide, a bioactive sphingolipid, has emerged as an important player in several cancers, including BC, due to its critical role in regulating both cell death and cell survival. In brief, intracellular accumulation of ceramides can induce cell death, while ceramides also serve as a substrate for the production of other sphingolipids that can promote cell survival and proliferation. Therefore, tumor cells tend to employ mechanisms to restrain ceramide levels while increasing the production of ceramide’s downstream sphingolipids to support growth. In contrast, BC treatment modalities can target the sphingolipid pathway to increase ceramide levels and ceramide-mediated cell death. Since ceramide is the common precursor of downstream pro-proliferative sphingolipids, it is important to have a global perspective of ceramide metabolism, both its synthesis and turnover, for a comprehensive evaluation of its role in BC [2,3,4,5].

In this review, we will highlight new developments over the last five years in (i) the role of ceramides in BC biology, (ii) the mechanisms by which ceramide levels are regulated in BC, and (iii) the therapeutic implications of ceramide production and metabolism in BC.

## 2. Ceramides: Structure and Production

**Figure 1:**
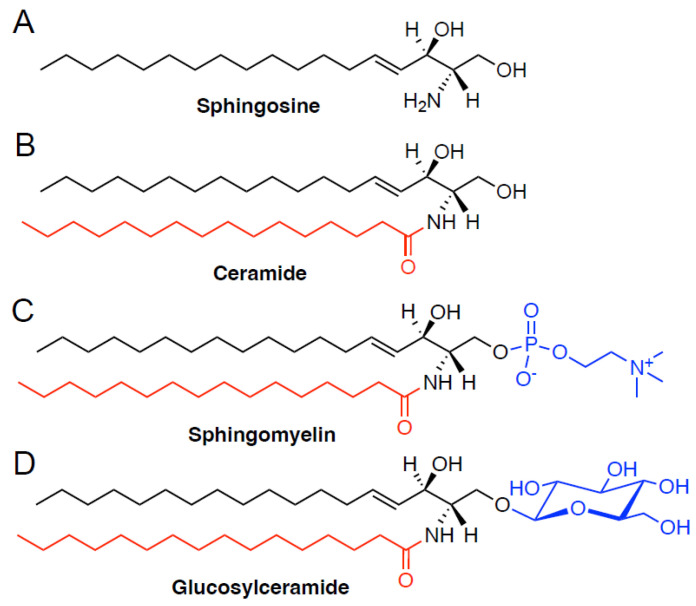
Structures of ceramides and other sphingolipids. (**A**) The most prevalent sphingoid base in mammals is a C18-sphingosine. General structures of ceramides (**B**), sphingomyelins (**C**) and glucosylceramides (**D**) on the C18-sphingoid backbone (black). Head groups of sphingomyelin and glucosylceramides are shown in blue. The acyl chain (red) length varies from 16 to 26 carbon-containing structures, predominantly in mammalian cells. C16 species are shown here as representative structures.

Ceramides are structurally defined as a sphingoid base, typically sphingosine with 18 carbons (d18), attached to a fatty acyl chain of variable length (14 to 26 carbons), the most common one being 16 carbons in mammalian cells (Figure 1) [6]. This characteristic amide group and the waxy nature of the molecules (‘*cer*’ meaning wax in Latin) give them the name ceramides. High hydrophobicity makes these molecules poorly water-soluble; therefore, they primarily exist in biological membranes. Ceramides are highly abundant in the outermost layer of our skin, making up about 30–40% of our epidermis and serving as a permeability barrier [7]. Intracellularly, ceramides are found in the plasma membrane, nuclear and mitochondrial envelope, endoplasmic reticulum (ER) and Golgi apparatus, where they carry out distinct functions. While ceramides in the plasma membrane serve in lipid rafts regulating membrane dynamics, ceramide accumulation in mitochondria induces apoptosis, and ceramides in the ER and Golgi are used as precursors to other sphingolipids [8].

**Figure 2:**
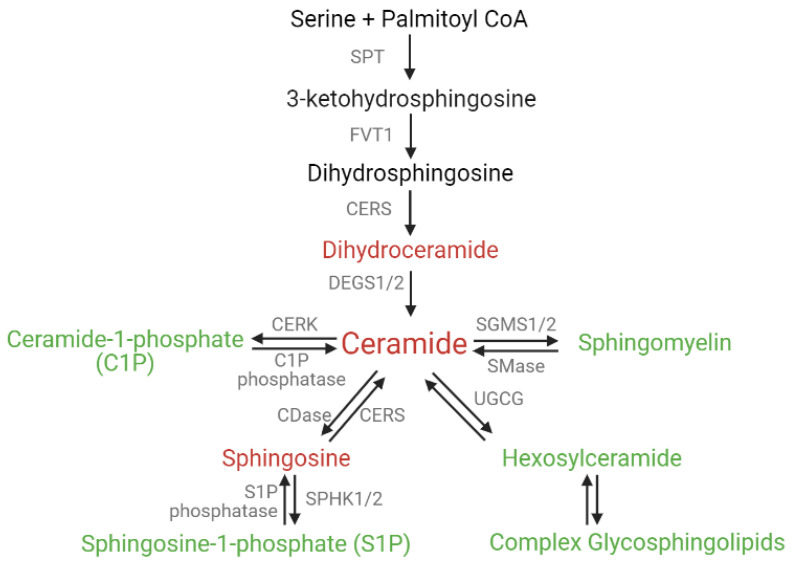
Ceramide production and turnover pathways. Enzymes are depicted in gray. Pro-apoptotic sphingolipids are depicted in red, and pro-proliferative sphingolipids are depicted in green. Diagram was created with Biorender.com.

Ceramides can be generated through de novo synthesis from the condensation of serine and a palmitoyl CoA at the ER by the enzyme serine-palmitoyl transferase (SPT). The product of this reaction is 3-keto dihydrosphingosine, which is reduced to dihydrosphingosine. Dihydrosphingosine is acylated by ceramide synthase (CERS) to yield dihydroceramide. Mammalian ceramide synthases are comprised of six isoforms, CERS1–6, which have substrate preferences based on acyl chain length. Dihydroceramides are then saturated by delta 4-desaturase (DEGS1/2) to produce ceramides. Ceramides can then be transported to the Golgi apparatus and be converted to downstream sphingolipids, which can also be converted back into ceramides and broken down through the sphingomyelinase and salvage pathways (Figure 2).

## 3. Canonical Role of Ceramides in BC: A Bona Fide Inducer of Cell Death

The role of ceramides in inducing apoptosis in different cancer cells, including BC, has been established by several lines of evidence. (i) Apoptosis-inducing agents increase intracellular ceramide levels prior to the initiation of the apoptotic cascade [9,10,11,12,13,14,15,16,17,18,19,20,21]. (ii) Intracellular delivery of ceramides and ceramide-analogs induces apoptosis [22,23,24,25,26]. (iii) Increasing endogenous ceramide levels trigger growth arrest and apoptosis [27,28,29,30,31,32]. Additionally, (iv) cell lines incapable of generating ceramides are resistant to chemo- and radiotherapy [17,22,27,33].

The current dogma about the mechanism by which ceramides induce apoptosis states that ceramides can form pores in the mitochondrial outer membrane (OMM), owing to their ability to form channels in planar phospholipid membranes [34]. Ceramide-induced pores in OMM result in an increased OMM permeability and a consequential release of cytochrome c and other mitochondrial proteins, such as SMAC/DIABLO, heat-shock proteins, and endonucleases, into the cytosol, thereby initiating the apoptotic cascade [35,36]. Consistent with this theory, reports show that OMM has very low ceramides and is enriched with dihydroceramides in healthy conditions. Dihydroceramides lack pore-forming ability due to their lack of a 4,5-trans bond as compared to their ceramide counterparts [37,38]. A recent report by Agnes De Mario and colleagues has suggested additional regulators that may control ceramide action in apoptosis. Ceramide-induced apoptosis in BC can be dependent on mitochondrial Ca^2+^ levels as an inhibitor of mitochondrial calcium uniporter (MCU), reducing mitochondrial Ca^2+^ uptake and decreasing Ca^2+^ load in the mitochondria, protecting BC cells from ceramide-induced apoptosis [39].

The fatty acyl chain length of ceramides can also be a critical factor in the molecular actions of ceramides. Several studies have described how short-chain and long-chain ceramides can have different biophysical properties that can affect their actions [40,41]. Increasing short-chain ceramides in breast cancer cells have been reported to reduce proliferation through inhibition of mTOR signaling in a recent study by Kim et al. [42]. In their study, overexpression of CERS6, which produces C14:0, C16:0, and C18:0 ceramides, but not other isoforms, resulted in inhibition of mTOR signaling and reduced cell proliferation in MCF-7 cells [42]. On the other hand, decreased levels of very long-chain ceramides (C20:0, C22:0, C24:0, and C26:0) have been reported to enhance proliferation and migration in luminal B breast tumors. Pani et al. reported that luminal B tumors have an alternate spliced (exon 8 skipped) CERS2 gene, which is associated with a poor prognosis of luminal B tumors [43]. The exon 8 corresponds to a segment in the catalytic domain of CERS2; hence, the alternate spliced variant becomes unable to synthesize long-chain ceramides, and the reduction of these ceramides promotes luminal B tumor growth. These findings suggest that more aggressive cancers are likely to employ regulatory mechanisms to restrain very long-chain ceramide generation from supporting cell proliferation and evading cell death.

Ceramide-mediated actions appear to be at the cornerstone of inducing apoptosis in BC cells. Over the last few years, several small molecules (such as fatostatin, hydroxytriolene, zoledronic acid, salvianolic acid, thymoquinone, etc.) have been described that promote cell death in BC cells through very different mechanisms [44,45,46,47,48]. For example, fatostatin induces ER-stress [44], and hydroxytriolene interacts with the plasma membrane, regulating its structure and composition, which reduces Akt signaling [45]. However, both of these small molecule inhibitors induce cell death by increasing the intracellular ceramide levels, albeit the detailed molecular underpinnings of how they increase ceramide levels have yet to be fully elucidated.

## 4. The Other Role of Ceramides: Conversion to Pro-Survival Sphingolipids

Ceramide, once transported to the Golgi apparatus, can also serve as a precursor to bioactive sphingolipids such as ceramide-1-phosphate (C1P) and sphingosine-1-phosphate (S1P), which can counteract the pro-apoptotic ceramide actions, or other sphingolipids such as sphingomyelin (SM) and hexosylceramide (HexCers) that are involved in cell survival, proliferation, and drug resistance (Figure 2). Ceramide conversion to other sphingolipids offers a plausible explanation for the detection of high ceramides in breast tumors from patients, as SM and S1P levels are also elevated in breast tumors [49,50,51,52,53]. Since ceramides are the precursors to produce the downstream sphingolipids, both ceramide de novo synthesis and ceramide turnover are increased in the breast tumor cells, as marked by increased gene expression of CERS2, -4, and -6, ceramide kinase (CERK), sphingosine kinase 1 (SPHK1), UDP-glucose ceramide glucosyltransferase (UGCG), and sphingomyelin synthase 1 (SGMS1), enzymes that are involved in ceramide turnover [49,53,54] (Table 1). Each of the downstream sphingolipids exerts a specific cellular function that can contribute to cell proliferation, metastasis, cancer stem cells, and drug resistance in different ways. Several studies in the past few years have improved our understanding of the cellular effects of ceramide turnover on different sphingolipids and may offer new targets for BC therapy.

### 4.1. Sphingosine-1-Phosphate (S1P)

Ceramide is converted to sphingosine by the action of ceramidases (CDase). Sphingosine is then phosphorylated by sphingosine kinase (SPHK1/2) to produce sphingosine-1-phosphate (S1P) [75]. S1P is secreted outside the cell, binds to S1P receptors (S1PR) and promotes cell proliferation and survival through activation of Akt and Erk-1/2 pathways [61,85]. Out of the five S1PR isoforms in humans, S1PR-1, -3, and -4 have been implicated in BC [86]. A recent study by Chen and colleagues has suggested that S1P can promote epithelial-mesenchymal transition (EMT) as well as stemness in BC cells [87]. S1P can also increase ceramide production and turnover in BC cells by increasing CERS1, -2, -6, and UGCG gene expression [88].

Three possible strategies to counter S1P’s ability to promote cell proliferation and survival have been tested in BC: (i) inhibition of CDase, (ii) inhibition of SPHK1/2 to prevent S1P production, and (iii) inhibition of S1P signaling. An inhibitor of CDase, D-erythro-MAPP treatment has been reported to increase intracellular ceramides and attenuate S1P generation, which induced cell death in MCF-7 cells (Table 1) [73]. Similarly, several small molecule inhibitors of SPHK1/2 and S1PR have been shown to inhibit BC cell growth both in vitro and in vivo [75,76,77,78,79,80], of which FTY720 (fingolimod, an FDA-approved drug for multiple sclerosis), has been extensively studied. FTY720 is a prodrug, which upon phosphorylation by SPHK2, yields phospho-FTY720, which acts as an antagonist for S1PR1, thereby inhibiting BC cell survival and proliferation [86]. Additionally, FTY720 has also been reported to potentiate the chemotherapeutic efficacy of docetaxel and doxorubicin in BC cells in two recent studies [81,82], thereby suggesting that attenuation of S1P generation or signaling can be a potential therapeutic strategy for the treatment of BC.

### 4.2. Ceramide-1-Phosphate (C1P)

Ceramide kinase (CERK) phosphorylates ceramides to produce ceramide-1-phosphate (C1P), another bioactive signaling sphingolipid involved in pro-survival and pro-proliferative actions. Recently, two studies have elucidated the molecular actions of C1P in BC, which suggested that cellular actions of C1P are mediated by the production of C-C Motif Chemokine Ligand 5 (CCL5) [89] and activation of PI3K and Akt pathways [90]. An increasing body of evidence has implicated C1P in cell migration and metastasis in BC [61,62,63]. A recent study that reported an increased CERK expression in the lung and bone metastatic cells of an MDA-MB-231 tumor supports the role of C1P in metastasis [90]. Along this line, CERK expression has been shown to be associated with a worse prognosis in TNBC patients [64]. A recent report by Zhu and colleagues has shown that overexpression of CERK in TNBC cells promotes cell growth, migration, and chemoresistance [65,66]. Interestingly, C1P also plays an important role in endocrine therapy-resistant cell survival, as inhibition of CERK induces cell death in therapy-resistant BC cells via loss of C1P [91]. Owing to C1P’s ability to inhibit de novo ceramide production, CERK inhibition can induce ceramide accumulation and consequent cell death in endocrine therapy-resistant BC cells [91,92].

Of note, high levels of very long odd-carbon chain C1P (C23:0 C1P and C23:1 C1P) have been detected in breast tumors from patients compared to the tumor-adjacent normal tissue [49], and C1P levels are also positively correlated with Ki-67 index of the breast tumors [49], suggesting that C1P level can be a potential prognostic parameter in breast cancer patients [93].

### 4.3. Sphingomyelins (SM)

Sphingomyelins (SM) are essential components of biological membranes; therefore, they are necessary for cell growth and proliferation. SM are generated from ceramides by the action of sphingomyelin synthase (SGMS-1/2). A recent report has implicated SM in BC metastasis and aggressiveness. Their findings suggest that SGMS2 promotes EMT through activation of the TGF-ß/SMAD pathway, more specifically, by increasing TGF-ß1 secretion [83]. An increase in ceramide turnover to SM is also an important feature of chemotherapy resistance in BC [84].

### 4.4. Hexosylceramides (HexCer)

From a wider perspective of overall cancers, the sphingolipids that play an important role in therapy resistance are undoubtedly the hexosylceramides (HexCer), a group of ceramide metabolites that have a neutral sugar moiety linked to a ceramide [94]. They serve as precursors to complex glycosphingolipids like globosides and gangliosides. The enzyme that converts ceramides to glucosylceramides, UDP-glucose ceramide glucosyltransferase (UGCG), is upregulated in multidrug resistance in multiple cancers [94,95]. In BC, UGCG has been reported to upregulate multidrug resistance protein 1 (MDR1), which confers drug resistance by acting as a drug-efflux pump, thereby keeping the intracellular drug concentration low [54]. An in vitro study has shown that co-suppression of MDR1 and UGCG can increase sensitivity to chemotherapeutic drugs in BC cells [96].

Glucosylceramides also play a crucial role in the metabolic reprogramming of breast cancer. Overexpression of UGCG increases glutamine synthesis and metabolism in BC, a common feature of therapy-resistant BC [67,68,69]. UGCG also confers additional metabolic changes which favor energy metabolism of therapy-resistance BC cells [70,71]. UGCG overexpression increases both glycolysis, oxidative phosphorylation, and amino acid synthesis in breast cancer cells [69,72]. In addition to metabolic changes, UGCG can also induce critical changes in the plasma membrane. Increasing glycosphingolipid and globotriacylceramide levels in the glycosphingolipid-enriched microdomains impacts multiple cellular signaling pathways in cell proliferation and drug resistance [97].

## 5. Therapeutic Implications of Ceramides in Breast Cancer

Treatment of BC involves multiple strategies, often depending on the molecular subtype and the size and spread of the tumor. While endocrine therapy (ET) (aromatase inhibitors or antiestrogens such as tamoxifen) is the standard of care for most hormone receptor-positive tumors, chemotherapy is the most common neoadjuvant therapeutic approach for other molecular types [98]. Depending on the presence of cancer cells in the sentinel node, radiotherapy is also often employed as a treatment modality [99]. Accumulation of ceramides has been reported as a result of ET, chemo- and radiotherapy, suggesting that ceramide-mediated cell death is an essential feature of neoadjuvant therapies, although ceramide accumulation occurs through different mechanisms. Tamoxifen treatment induces ceramide accumulation and consequent cell death in MCF-7 and MDA-MB-231 cells through the inhibition of acid ceramidase (aCDase) [58,59,100]. Tamoxifen-mediated inhibition of aCDase and the subsequent increase in ceramides and loss of S1P are thought to occur in an estrogen receptor-independent manner, suggesting tamoxifen may have some efficacy in in triple negative breast cancer, as well as other cancer types [101].

In contrast to ET, ionizing radiation relies on acid sphingomyelinase (aSMase) activity to induce ceramide accumulation and cell death in BC cells [57,60]. This was supported by a study where aSMase-null lymphoblasts were shown to be insensitive to radiation therapy and to be re-sensitized upon aSMase overexpression [102]. Similar to radiotherapy, cellular actions of chemotherapeutic agents, such as paclitaxel, also involve aSMase-mediated ceramide generation and subsequent cell death in BC cells [103]. Additionally, another study has reported that paclitaxel can also increase de novo ceramide production through activating SPT in breast tumors [55], suggesting that chemotherapeutic agents may promote intracellular ceramide accumulation through a combination of increasing de novo ceramide production and breakdown of downstream sphingolipids.

Considering the role of ceramides in cell death and the role of ceramide downstream metabolites in cell proliferation and survival, it is plausible that therapy-resistant cells take certain measures to keep their ceramide levels regulated. Recently, Shammout and colleagues compared doxorubicin-sensitive and -resistant MCF-7 cells and found that the doxorubicin-resistant cells maintain an increased level of SM and decreased levels of ceramides, dihydroceramides, and HexCers [84]. Similarly, our profiling study of tamoxifen-sensitive and -resistant cells also found decreased ceramide and HexCers levels in tamoxifen-resistant cells, although SM and dihydroceramide levels were found to be unaltered [91]. The different sphingolipidomic changes employed by different therapy-resistant cells to maintain lower ceramide levels need to be validated in preclinical models and patient tumors. Additionally, ceramide downregulation also requires to be mechanistically elucidated for devising new therapeutic strategies and improving patient outcomes.

The majority of the ceramide-based therapeutics in BC are in preclinical or clinical I/II phases and are mostly focused on preventing ceramide turnover to downstream sphingolipids (Table 2). Few studies that have attempted to deliver ceramides to tumor cells to induce cell death have used synthetic short-chain ceramides formulated in nanoliposomes for an efficacious intracellular delivery. Ceramide nanoliposomes (CNL) have inhibited cell proliferation and migration in TNBC cells [58,104,105]. Of note, one study has employed a topical application of C2 and C6 CNL in a phase II study against cutaneous breast cancer patients. While the formulation of C2 and C6 CNL showed no toxicity in patients, only 4% responded to the treatment [106]. Although this low response rate in patients suspended further clinical trials with CNL, adding short-chain ceramides in nanoliposome-based formulations has been studied to increase the chemotherapeutic efficacies BC drugs [107,108,109]. Of note, C12-ceramide-containing liposomes have improved cellular targeting and synergized with the therapeutic efficacy of docetaxel and doxorubicin in BC cells in a recent study [110].

## 6. Concluding Remarks

Ceramides have become increasingly relevant in BC with a growing understanding of the different regulatory mechanisms of ceramide production and turnover employed by the cancer cells to promote different hallmarks of cancer. The understanding of the regulatory network has also been essential for the development of new therapeutic strategies for BC patients. Several ceramide-based cancer therapeutics are currently being tested in preclinical and clinical (phase I and II) trials for BC, either alone or in combination with other neoadjuvant therapies [138].

Of note, sphingolipidomic profiling studies of therapy-sensitive and -resistant tumors have opened the possibility of ceramide-based treatment modalities in therapy-resistant breast tumors. In both chemotherapy and endocrine therapy-resistant BC cells, maintaining low ceramide levels has been observed as a common feature of therapy resistance. However, these observations are yet to be validated in patient-derived xenografts (PDX) or patient tumors. Further evidence and more mechanistic knowledge about this altered ceramide regulation can potentially be leveraged into an improved therapeutic application for patients with therapy-resistant disease.

There are several pertinent questions that remain to be addressed. For example, the genetic determinants for altered ceramide regulation are largely unresolved. Apart from a recent study showing alternative splicing of CERS2 in luminal B tumors, genetic signatures for altered ceramide regulation are not well-elucidated. Additionally, the cellular and molecular determinants for ceramide sensitivity are also somewhat obscure. As a bioactive lipid, ceramide can interact with other proteins, and these interactions in BC need to be characterized. Future studies elucidating these mechanisms will offer novel and improved strategies and a new frontier of BC therapeutics.

## Figures and Tables

**Table 1:** List of enzymes and their inhibitors of the ceramide synthesis and turnover pathways.

Enzyme Name (Abbreviation)	Gene Name(s)	Actions	Major Implication(s) in BC	Inhibitor	Citations
Serine palmitoyl transferase (SPT)	SPTLC1–3, SPTSSA-B	De novo ceramide synthesis	Enzyme activity increases in response to chemo- and radiotherapy		[55]
Ceramide synthase	CERS1	C18:0, C20:0 ceramide synthesis	Ceramide production under different stimulus	FB1	[56]
	CERS2	C20:0, C22:0, C24:0, C26:0 ceramide synthesis	Long-chain ceramide production; alternative splicing drives aggressive luminal B phenotype		[43]
	CERS3	C16:0, C18:0, C22:0, C24:0 ceramide synthesis	Ceramide production under different stimulus		[44,45,46,47,48,49,56,57]
	CERS4	C18:0, C20:0, C22:0, C24:0, C26:0 ceramide synthesis			
	CERS5	C14:0, C16:0 C18:0, C18:1 ceramide synthesis			
	CERS6	C14:0, C16:0, C18:0 ceramide synthesis	Short-chain ceramide production; inhibits cell proliferation through mTOR pathway.		[42]
Sphingomyelinase (SMase)	SMPD2	Ceramide production	Induce cell cycle arrest	GW4869	[31,32]
	SMPD1	Ceramide production	Activity is required for chemo and radiotherapy		[58,59,60]
Ceramide kinase	CERK	C1P generation	Cell migration and metastasis	NVP-231	[61,62,63,64,65,66]
UDP-glucose ceramide glucosyltransferase	UGCG	Glucosylceramide generation	Metabolic reprogramming, increased energy metabolism		[67,68,69,70,71,72]
Acid Ceramidase	ASAH1	Sphingosine production and subsequent S1P production	S1P generation for promoting BC growth	D-erythro-MAPP	[73,74]
Sphingosine kinase	SPHK1/2	S1P generation	BC growth and proliferation	FTY720	[75,76,77,78,79,80,81,82]
Sphingomyelin synthase	SGMS1/2	SM generation	Promoting EMT, metastasis and chemoresistance		[83,84]

**Table 2:** List of ceramide-based therapeutics in preclinical and clinical studies in breast cancer.

Drug/Compound Name	Target	Combination	Phase	Citations
Fingolimod (FTY720)	Structural analog of sphingosine, S1PR antagonist	Alone	Preclinical	[111,112,113,114,115]
		Sunitinib malate	Preclinical	[116]
		Radiation	Preclinical	[117]
		Doxorubicin	Preclinical	[118]
		Cisplatin	Preclinical	[119]
Fenretinide	Inhibit DEGS1/2	Alone	Preclinical	[120]
		Alone	Phase I/II	[121,122,123,124]
		Tamoxifen	Phase I/II	[125,126,127,128,129]
Safingol	Inhibit SPHK1	Alone	Preclinical	[130,131]
ABC294640	Inhibit SPHK2 and DEGS1	Alone	Preclinical	[132,133,134]
Ceramide- nanoliposomes (CNL)	Ceramide delivery	Alone	Preclinical	[104,105,106]
		Tamoxifen	Preclinical	[58]
SKI-II	SPHK1/2 inhibitor	Alone	Preclinical	[78,135]
α-GalCer	Synthetic glycolipid α-galactosyl ceramide, a strong immunostimulant	Alone	Preclinical	[136,137]

## Data Availability

Not applicable.
